# Reducing cognitive arousal and sleep effort alleviates insomnia and depression in pregnant women with DSM-5 insomnia disorder treated with a mindfulness sleep program

**DOI:** 10.1093/sleepadvances/zpad031

**Published:** 2023-08-05

**Authors:** David A Kalmbach, Philip Cheng, Anthony N Reffi, Jason C Ong, Leslie M Swanson, Colin A Espie, Grace M Seymour, Mika Hirata, Olivia Walch, D’Angela S Pitts, Thomas Roth, Christopher L Drake

**Affiliations:** Thomas Roth Sleep Disorders & Research Center, Henry Ford Health, Detroit, MI, USA; Department of Obstetrics, Gynecology, and Reproductive Biology, Michigan State University College of Human Medicine, East Lansing, MI, USA; Thomas Roth Sleep Disorders & Research Center, Henry Ford Health, Detroit, MI, USA; Thomas Roth Sleep Disorders & Research Center, Henry Ford Health, Detroit, MI, USA; Center for Circadian and Sleep Medicine, Department of Neurology, Northwestern University Feinberg School of Medicine, Chicago, IL, USA; Behavioral Sleep Medicine, Nox Health, Suwanee, GA, USA; Department of Psychiatry, University of Michigan, Ann Arbor, MI, USA; Nuffield Department of Clinical Neurosciences, Oxford University, Oxford, UK; Big Health, San Francisco, CA, USA; Thomas Roth Sleep Disorders & Research Center, Henry Ford Health, Detroit, MI, USA; Thomas Roth Sleep Disorders & Research Center, Henry Ford Health, Detroit, MI, USA; Department of Neurology, University of Michigan, Ann Arbor, MI, USA; Arascope Inc, Falls Church, VA, USA; Department of Obstetrics, Gynecology, and Reproductive Biology, Michigan State University College of Human Medicine, East Lansing, MI, USA; Maternal Fetal Medicine, Henry Ford Health, Detroit, MI, USA; Thomas Roth Sleep Disorders & Research Center, Henry Ford Health, Detroit, MI, USA; Thomas Roth Sleep Disorders & Research Center, Henry Ford Health, Detroit, MI, USA

**Keywords:** PUMAS, worry, rumination, pregnancy, meditation, stress, MBI, MBSR, mechanism

## Abstract

**Objectives:**

Combining mindfulness with behavioral sleep strategies has been found to alleviate symptoms of insomnia and depression during pregnancy, but mechanisms for this treatment approach remain unclear. The present study examined nocturnal cognitive arousal and sleep effort as potential treatment mechanisms for alleviating insomnia and depression via a mindfulness sleep program for pregnant women.

**Methods:**

Secondary analysis from a proof-of-concept trial of 12 pregnant women with DSM-5 insomnia disorder who were treated with Perinatal Understanding of Mindful Awareness for Sleep (PUMAS), which places behavioral sleep strategies within a mindfulness framework. Data were collected across eight weekly assessments: pretreatment, six sessions, and posttreatment. Measures included the insomnia severity index (ISI), Edinburgh postnatal depression scale (EPDS), pre-sleep arousal scale’s cognitive factor (PSASC), and the Glasgow sleep effort scale (GSES). We used linear mixed modeling to test cognitive arousal and sleep effort as concurrent and prospective predictors of insomnia and depression.

**Results:**

Most patients reported high cognitive arousal before PUMAS (75.0%), which decreased to 8.3% after treatment. All insomnia remitters reported low cognitive arousal after treatment, whereas half of nonremitters continued reporting high cognitive arousal. Both nocturnal cognitive arousal and sleep effort were associated with same-week changes in insomnia throughout treatment, and sleep effort yielded a prospective effect on insomnia. Lower levels of nocturnal cognitive arousal and sleep effort prospectively predicted reductions in depression.

**Conclusions:**

The present study offers preliminary evidence that reducing sleep effort and nocturnal cognitive arousal may serve as key mechanisms for alleviating insomnia and depression via mindfulness-based insomnia therapy. ClinicalTrials.gov ID: NCT04443959

**Statement of Significance**

Perinatal Understanding of Mindful Awareness for Sleep (PUMAS, which combines behavioral sleep strategies with mindfulness) has efficacy for prenatal insomnia and comorbid depression. The present study sought to elucidate treatment mechanisms of PUMAS. Our trial showed that reducing sleep and cognitive arousal led to alleviations in insomnia and depression. We theorize that PUMAS targets cognitive arousal and sleep effort via two paths wherein (1) mindfulness reduces perseverative thinking and cognitive effort to force sleep at night while (2) sleep restriction reduces sleep effort and cognitive arousal by maximizing sleep pressure at bed-time. Combining mindfulness and behavioral sleep strategies may dually target cognitive arousal and sleep effort by fostering a calm mindset at night when sleep pressure is behaviorally maximized.

## Introduction

Insomnia affects half of pregnant women with 20% of all pregnant women meeting criteria for DSM-5 insomnia disorder [1–3]. Cognitive-behavioral therapy for insomnia (CBTI) is efficacious during pregnancy and has durable benefits into postpartum [4–10]. Although clinical trial results are mixed, CBTI appears to modestly alleviate perinatal depression, which is a key benefit as half of pregnant women with insomnia endorse comorbid depression [2, 11, 12]. Despite promising results, CBTI undertreats cognitive arousal during pregnancy, which may limit its efficacy [13–15].

Cognitive arousal—heightened cognitive activity including worry and rumination—is central to insomnia etiology and maintenance [16–20]. A salient manifestation of cognitive arousal in insomnia is heightened sleep effort [20, 21]. Unlike the effortlessness of normal sleep, people with insomnia often exert high sleep effort, characterized by active cognitive effort to force sleep and excessive preoccupation about sleep, which—despite intentions—disrupts sleep [19–21]. Pregnant women whose cognitive arousal does not improve with CBTI are three times less likely to remit from insomnia than those whose arousal decreases (23% vs 77% remission; unreported results from our RCT [9, 14]). Although CBTI produces mixed findings for cognitive arousal in the nonperinatal population [22–25], evidence nevertheless suggests that reducing cognitive arousal can facilitate alleviation of insomnia and depression [22, 23]. Consequently, researchers have proposed that enhancing insomnia therapy effects on cognitive arousal may improve efficacy for insomnia and comorbid depression during pregnancy [13, 15, 26, 27].

Mindfulness-based interventions (MBIs) substantially reduce cognitive arousal [28–30]. Moreover, combining mindfulness with behavioral sleep strategies produces large clinical benefits for sleep and cognitive arousal [27, 30–32]. To address cognitive arousal directly in prenatal insomnia, we developed Perinatal Understanding of Mindful Awareness for Sleep (PUMAS), which places behavioral sleep strategies within a MBI framework tailored to pregnancy. PUMAS was designed to foster a calm mindset at night via mindfulness exercises and skills while behaviorally maximizing sleep pressure at bedtime via sleep restriction and stimulus control. In our proof-of-concept trial of 12 women with DSM-5 insomnia disorder, PUMAS produced large reductions in insomnia (83.3% remission), depression, cognitive arousal, and sleep effort [33].

The present study was a secondary analysis of our proof-of-concept trial. We sought to better understand the interplay among insomnia, depression, cognitive arousal, and sleep effort. Specifically, we evaluated nocturnal cognitive arousal and sleep effort as potential treatment mechanisms in PUMAS. We examined data from 12 patients who provided up to eight weeks of assessments spanning pretreatment to posttreatment. We hypothesized: (1) that weekly levels of insomnia and depression would be associated with same-week levels of nocturnal cognitive arousal and sleep effort; (2) lower levels of cognitive arousal and sleep effort would prospectively predict decreased insomnia and depression a week later.

## Methods

### Participants and procedures

This study was approved by IRB and conducted at Henry Ford Health (HFH) in Metro Detroit, Michigan, USA. Participants provided informed consent. Intent-to-treat (ITT) analyses supported PUMAS efficacy for insomnia, depression, nocturnal cognitive arousal, and sleep effort [33]. This secondary analysis characterizes mechanisms facilitating alleviation of insomnia and depression.

**Figure 1.**
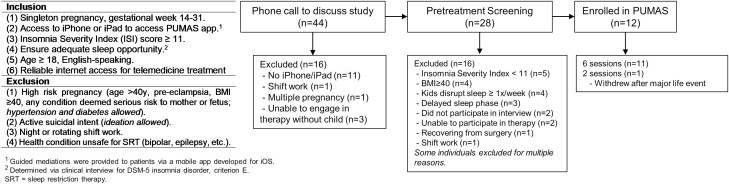
Eligibility criteria and CONSORT flow diagram.

Briefly, we screened 28 treatment-seeking women and enrolled 12 patients with DSM-5 insomnia disorder (Figure 1 for eligibility criteria). Although diagnosis was not required, all patients were interviewed to determine DSM-5 insomnia disorder status. See Figure 1 for CONSORT flow diagram. See published report for methodological details [33].

### Study intervention

*PUMAS* was delivered via six weekly 60-minute video sessions in a 1:1 therapist to patient format. Providers included two clinical psychologists and a registered nurse. Influenced by mindfulness-based therapy for insomnia (MBTI) [30, 31, 34], PUMAS places behavioral sleep strategies (sleep restriction, stimulus control) in a MBI framework. All components are tailored to pregnancy (e.g., meditations include the baby). See Kalmbach et al. (2023) for details [33].

### Measures

Assessments were collected via online surveys (Qualtrics). All surveys were completed on weekdays, which was not by design, but was observed after data collection. Sociodemographics and perinatal health were assessed at pretreatment screening. Study outcomes were assessed at eight weekly time-points: pretreatment screening, the day of each treatment session (survey completed *before* each session), and posttreatment assessment. The *Insomnia Severity Index (ISI)* is validated in pregnancy [2, 35]. ISI ≥ 11 detects DSM-5 insomnia disorder during pregnancy [2] and was required for study inclusion. Insomnia remission was operationalized as ISI ≤ 7 [2, 35]. The *Edinburgh Postnatal Depression Scale (EPDS)* measured depression [36]. We operationalized EPDS ≥ 10 as a positive depression screen, which is the original cutoff for detecting major or minor depression, and aligns with practice guidelines in HFH women’s health clinics for detecting perinatal depression. The *Pre-Sleep Arousal Scale’s Cognitive factor (PSASC)* measured nocturnal cognitive arousal and is validated in pregnancy [2, 37]. PSASC ≥ 18 reflects high cognitive arousal. The *Glasgow Sleep Effort Scale (GSES)* measured sleep effort, which assesses preoccupation with sleep and cognitive effort to force sleep [21].

### Analysis Plan

Descriptive analyses were performed in SPSS 26 (IBM) and linear mixed modeling was performed in STATA 15.1 (StataCorp). We tested hypotheses using linear mixed models to examine within-subjects effects using an ITT approach. We first regressed repeated measures of the clinical outcome (insomnia, depression) on concurrent levels of nocturnal cognitive arousal and sleep effort while controlling for time and other clinical symptoms. These models described whether weekly levels of insomnia/depression were associated with weekly levels of cognitive arousal and sleep effort. To test prospective effects, clinical outcomes (insomnia, depression) were regressed on lagged values (by one week) of nocturnal cognitive arousal and sleep effort while controlling for time and the other clinical outcome (e.g., control for depression when predicting insomnia). Lagged models showed prospective effects of nocturnal cognitive arousal and sleep effort on clinical outcomes (e.g., does sleep effort predict insomnia a week later?). For descriptive purposes, we conducted posthoc chi-square analyses comparing rates of insomnia remission among patients with high vs low nocturnal cognitive arousal.

## Results

### Patient characteristics

At enrollment, all patients were in the 2^nd^ trimester and met diagnostic criteria for DSM-5 insomnia disorder and reported ISI scores ≥ 11. Five patients (41.7%) screened positive for depression (EPDS ≥ 10). After PUMAS, 10 patients (83.3%) remitted from insomnia and no patients screened positive for depression. The sample was predominantly non-Hispanic white (75.0%), highly educated (83.3% college degree or higher), and nulliparous (66.7%). See Table 1 for sample characteristics.

**Table 1.** Patient characteristics (*n* = 12).

Age (*M* ± SD, range)	30.33 ± 4.23, 22-36 years	Education level	
Gestational week (*M* ± SD, range)	23.67 ± 1.97, 21-27 weeks	Some college credit, but no degree	*n* = 1/12, 8.3%
Relationship status (*n*, %)		Trade, technical, or vocational certificate	*n* = 1/12, 8.3%
Married	*n* = 11/12, 91.7%	Bachelor’s degree	*n* = 6/12, 50.0%
Not married, but in a relationship	*n* = 1/12, 8.3%	Master’s degree	*n* = 4/12, 33.3%
Planned pregnancy (n, %)	*n* = 9/12, 75.0%	Employment status (n, %)	
Nulliparous (n, %)	*n* = 8/12, 66.7%	Full-time	*n* = 8/12, 66.7%
Other kids at home	*n* = 5/12, 41.7%	Part-time	*n* = 2/12, 16.7%
BMI (M ± SD, range)	29.98 ± 4.50, 21.80-39.00	Stay-at-home parent or homemaker	*n* = 2/12, 16.7%
BMI ≥ 35 (n, %)	*n* = 1/12, 8.3%	Annual household income (n, %)	
Hypertension (n, %)		< $20,000	*n* = 3/12, 25.0%
Gestational onset	*n* = 1/12, 8.3%	$75,001 - $100,000	*n* = 6/12, 50.0%
Pre-gestational onset	*n* = 1/12, 8.3%	$100,001 - $200,000	*n* = 2/12, 16.7%
Diabetes, type II (n,%)	*n* = 0/12, 0.0%	> $200,000	*n* = 1/12, 8.3%
Race (n, %)		DSM-5 insomnia disorder (n, %)	
white	*n* = 9/12, 75.0%	Chronic: Met criteria, duration ≥ 3 months	*n* = 11/12, 91.7%
Black	*n* = 1/12, 8.3%	Episodic: Met criteria, duration = 2 months	*n* = 1/12, 8.3%
East or Southeast Asian	*n* = 1/12, 8.3%	Sleep duration (M ± SD)	6.67 ± .96 hours
Multi-racial	*n* = 1/12, 8.3%	≤ 6 hours/night (n, %)	*n* = 3/12, 25.0%
		Habitual snoring (≥ 3 nights/week; n, %)	*n* = 2/12, 16.7%
		Antidepressant medication (n, %)	*n* = 1/12, 8.3%
		Sleep aids (n, %)	*n* = 0/12, 0.0%

M ± SD, mean and standard deviation; BMI, body mass index.

**Figure 2.**
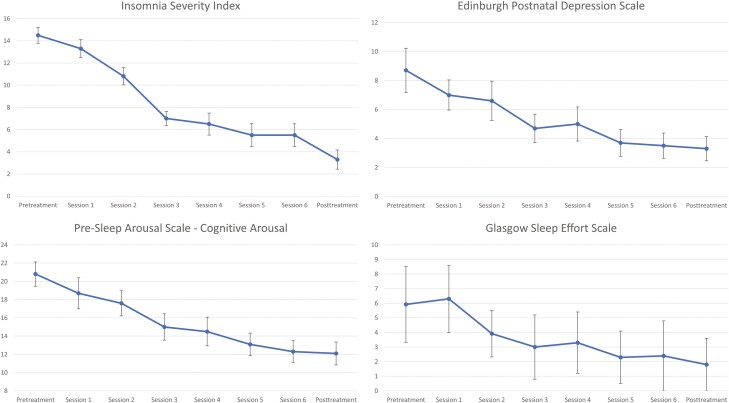
Means and Standard Errors presented for each measure (ISI, EPDS, PSASC, and GSES) across each time-point. Data are presented for descriptive purposes.

We collected 92 observations across 12 patients. Eleven women completed all six sessions and all eight assessments. One patient completed two sessions and four assessments (pretreatment, two weekly surveys, then an abbreviated posttreatment) before withdrawing due to a major life event. See Figure 2 for weekly levels of symptoms across assessments.

THE FIGURE'S AXIS LABELS ARE EXTREMELY SMALL; IS IT POSSIBLE TO ENLARGE THEM?

### Do cognitive arousal and sleep effort predict change in insomnia symptoms?

We first used linear mixed modeling to regress weekly ISI on same-week levels of PSASC and GSES while controlling for time and EPDS. Results showed that ISI decreased over time, and that weekly levels of ISI were associated with both same-week levels of PSASC and GSES (Table 2).

**Table 2.** Linear mixed models regressing insomnia/depression on nocturnal cognitive arousal and sleep effort.

*Within-subjects factors*	*b*	*95%CI*	*Z*	*p*
Predicting concurrent ISI: Wald χ^2^ = 273.55, p < .001 (n = 12, obs = 92).				
Time	−0.97	−1.25, −0.68	−6.63	<.001
PSASC	0.25	0.06, 0.44	2.62	.009
GSES	0.40	0.04, 0.77	2.18	.029
EPDS	0.06	−0.17, 0.29	0.52	.602
Predicting future ISI: Wald χ^2^ = 128.97, p < .001 (n = 12, obs = 80).				
Time	−0.93	−1.39, −0.47	−3.94	<.001
PSASC, lagged 1 week	−0.01	−0.24, 0.20	−0.13	.896
GSES, lagged 1 week	0.51	0.10, 0.92	2.45	.01
EPDS, lagged 1 week	−0.01	−0.27, 0.25	−0.06	.955
ISI, lagged 1 week	0.15	−0.09, 0.38	1.24	.216
Predicting concurrent EPDS: Wald χ^2^ = 109.24, *p* < .001 (*n* = 12, obs = 92).				
Time	−0.12	−0.42, 0.18	−0.79	.429
PSASC	0.30	−014, 0.45	3.68	<.01
GSES	0.21	−0.11, 0.53	1.31	.191
ISI	0.08	−0.10, 0.25	0.84	.401
Predicting future EPDS: Wald χ^2^ = 82.84, *p* < .001 (*n* = 12, obs = 80).				
Time	−0.27	−0.57,.04	−1.73	.083
PSASC, lagged 1 week	0.28	0.13,.42	3.75	<.001
GSES, lagged 1 week	0.42	0.14,.70	2.94	.003
ISI, lagged 1 week	−0.17	−0.33,.01	−2.07	.038
EPDS, lagged 1 week	−0.04	−0.24,.16	−0.41	.682

ISI, insomnia severity index; EPDS, Edinburg postnatal depression scale; PSASC, pre-sleep arousal scale’s cognitive factor; GSES, Glasgow sleep effort scale; Wald χ^2^ reflects the difference between the tested model and a null model with no predictors. Thus, a significant chi-square is desirable as it indicates a model that accounts for significant variance in the outcome. obs = observations. b = parameter estimate. 95% CI = 95% confidence interval for the coefficient. z = z-statistic. p = significance value.

To test prospective effects, we regressed weekly ISI on lagged values of PSASC and GSES while controlling for covariates (Table 2). GSES scores predicted next-week ISI such that lower levels of sleep effort predicted decreased insomnia one week later. Once again, ISI decreased significantly over time. No other predictors were significant.

#### Posthoc: Cognitive arousal and insomnia remission.

Before PUMAS, 75.0% of patients screened positive for high cognitive arousal (PSASC ≥ 18). Notably, pretreatment cognitive arousal status did not predict insomnia remission (ISI ≤ 7; χ^2^ = 0.48, *p* = .488). After PUMAS, only one patient (8.3%) reported high cognitive arousal. Moreover, posttreatment cognitive arousal status and insomnia remission were significantly related such that 0.0% of remitters reported high cognitive arousal after PUMAS (*n* = 0/10), whereas half of non-remitters reported high arousal after treatment (n = 1/2; χ^2^ = 5.46, *p* = .020).

### Do cognitive arousal and sleep effort predict change in depression?

Linear mixed modeling showed that weekly EPDS scores were associated with PSASC such that lower levels of cognitive arousal were associated with same-week lower levels of depression (Table 2).

To test prospective effects, we regressed weekly EPDS on lagged values of PSASC and GSES while controlling for covariates (Table 2). Results showed that both PSASC and GSES predicted next-week EPDS such that lower levels of cognitive arousal and sleep effort predicted decreased depression the following week. Lagged ISI predicted EPDS, but this association was erroneous.^1^

## Discussion

The present study evaluated potential treatment mechanisms of a mindfulness-based sleep program (PUMAS) in 12 pregnant women with DSM-5 insomnia disorder. Prospective data preliminarily supported reductions in nocturnal cognitive arousal and sleep effort as candidate mechanisms for alleviating insomnia and depression.

### Reducing cognitive arousal and sleep effort may alleviate insomnia and depression

#### Insomnia.

In the non-perinatal population, reducing cognitive arousal facilitates reduction of insomnia [22, 23]. In the present trial, weekly reductions in nocturnal cognitive arousal were associated with same week reductions in insomnia severity. Critically, all patients who remitted from insomnia reported low levels of cognitive arousal after PUMAS, whereas half of nonremitters rated their arousal as high after treatment. Important to emphasize is that pretreatment cognitive arousal did not predict remission. This finding is consistent with prior RCT data in pregnant women suggesting that refractory cognitive arousal (i.e., cognitive arousal that does not alleviate despite treatment) is a barrier to treatment success, whereas pretreatment arousal does not predict therapy response [14].

Our findings also expand on prior knowledge by indicating that sleep effort—a manifestation of high cognitive arousal marked by excessive preoccupation about sleep and increased cognitive effort to force sleep—may serve as a treatment mechanism in prenatal insomnia therapy. Weekly reductions in sleep effort were associated with concurrent and prospective reductions in insomnia, thereby suggesting that the sleep effort aspect of cognitive arousal is an important target for alleviating prenatal insomnia.

#### Depression.

Prospective data show that reducing nocturnal cognitive arousal and sleep effort leads to alleviations in depression for PUMAS patients. As efficacy data suggest that PUMAS yields large reductions in cognitive arousal and sleep effort [33], the present study empirically supports recommendations to more directly address cognitive arousal in prenatal insomnia therapy to enhance antidepressant effects [13, 15, 26, 27].

### Combining mindfulness with behavior sleep strategies may be well-suited to address cognitive arousal and sleep effort

MBIs have a well-documented effect on cognitive arousal [28, 30, 38, 39]. Moreover, obviating sleep effort by maximizing nighttime sleep pressure is a proposed a mechanism of sleep restriction [40–42]. Thus, treatment approaches combining behavioral sleep strategies with mindfulness may be well-suited to address cognitive arousal and sleep effort at night.

Indeed, PUMAS reduces nocturnal cognitive arousal and sleep effort [33], which aligns with RCT data from nonperinatal insomnia patients treated with MBTI [30]. As an MBI, PUMAS leverages guided meditations, engagement in mindful activities, and present-moment awareness of pleasant and unpleasant events to apply mindfulness principles to sleep, stress regulation, and daily life. Importantly, reducing mind-wandering and intrusive thoughts is a central tenet of PUMAS, and focus group data from poorly sleeping pregnant women identified reducing worry as key to improving prenatal sleep [43].

**Figure 3.**
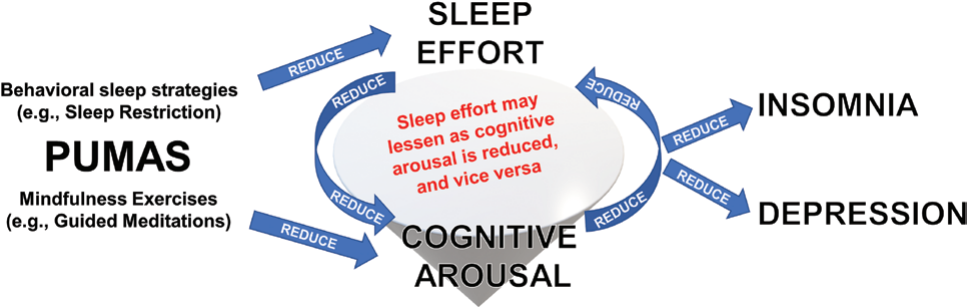
Theoretical framework for PUMAS treatment mechanisms for alleviating insomnia and depression. Path 1: Behavioral sleep strategies (e.g., sleep restriction) maximize nighttime sleep pressure, which obviates sleep effort. Path 2: Mindfulness exercises (e.g., meditations, especially at night) reduce perseverative thinking and cognitive effort to force sleep at night. Taken together, behavioral sleep strategies and mindfulness may dually target cognitive arousal and sleep effort by fostering a calm mindset at night when sleep pressure is behaviorally maximized. Reducing sleep effort and cognitive arousal alleviate insomnia and depression symptoms.

We theorize that PUMAS may target cognitive arousal and sleep effort via two paths (Figure 3). First, mindfulness exercises and skills (e.g., guided meditations, applying non-striving to sleep) are designed to reduce mind-wandering (including affectively charged cognitive preoccupations like worry and rumination) and sleep effort at night. Second, sleep restriction (a behavioral component of PUMAS, MBTI, and CBTI) may reduce sleep effort and cognitive arousal by maximizing sleep pressure at bed-time [40, 42, 44]. Thus, combining mindfulness and behavioral sleep strategies—like PUMAS and MBTI—may dually target cognitive arousal and sleep effort by fostering a calm mindset at night when sleep pressure is behaviorally maximized.

FIGURE 3 SHOULD BE CORRECTED FOR THE ONE ARROW WHERE REDUCE IS UPSIDE DOWN

### Limitations

Results should be interpreted considering methodological limitations. First, although our 92 observations lend adequate power to detect within-subjects effects, the weekly assessments schedule may not be optimal for capturing effects of reducing cognitive arousal/sleep effort on sleep. Rather, effects of cognitive arousal and sleep effort occur *within a night* (I ruminate tonight, so I have difficulty falling asleep tonight). A recent pregnancy study showed that cognitive arousal captured at night is a more robust predictor of nocturnal wakefulness than ratings of cognitive arousal over the prior week [45]. Second, increasing mindfulness and decentering are proposed mechanisms in MBIs [46–48], but were not assessed weekly in this study. Third, the sample was mostly non-Hispanic white and highly educated. Replication is needed in a more diverse, representative sample. Fourth, a lack of control group is a limitation. The present study was a proof-of-concept trial to evaluate the preliminary efficacy, patient uptake, and potential mechanisms of this newly developed program. We believe results from this study and our prior report [33] support conducting a RCT, which would allow for a more rigorous mechanistic test. Finally, we were underpowered to detect between-subjects effects, thus their exclusion as a main focus of our report. Along these lines, post hoc rates of high/low cognitive arousal among remitters and non-remitters were reported for descriptive purposes only. Our small sample size limits generalizability of these rates and they should not be considered population estimates.

### Conclusions and future directions

Reducing nocturnal cognitive arousal and sleep effort may be key treatment mechanisms for alleviating insomnia and depression in pregnant women with DSM-5 insomnia disorder. However, these potential mechanisms need to be replicated in a RCT. Therapy approaches combining mindfulness and behavioral strategies may be uniquely well-suited for mitigating the insomniogenic and depressogenic effects of cognitive arousal and sleep effort.

## Footnotes

1 The temporal association of high ISI predicting low EPDS was interpreted as erroneous for several reasons: (1) The negative direction runs counter to a very well-established literature. (2) The significance of the association runs counter to null results regarding an association between ISI and EPDS in the three previous models included in this report, thus the significant result here is unreliable and not replicated within this study. (3) ISI, PSASC, and GSES are highly correlated (*r* = .64 to *r* = .77). When PSASC and GSES are NOT included in the model, lagged ISI is not significant (*p* = .747). Moreover, when only PSASC or GSES is removed from this lagged model, then PSASC/GSES remains significant, but ISI is not significant. Taken together, this significant association is clearly erroneous and does not warrant further interpretation.
